# Oxytocin, but not vasopressin, decreases willingness to harm others by promoting moral emotions of guilt and shame

**DOI:** 10.1038/s41380-024-02590-w

**Published:** 2024-05-20

**Authors:** Xiaoxiao Zheng, Jiayuan Wang, Xi Yang, Lei Xu, Benjamin Becker, Barbara J. Sahakian, Trevor W. Robbins, Keith M. Kendrick

**Affiliations:** 1 The Center of Psychosomatic Medicine, Sichuan Provincial Center for Mental Health, Sichuan Provincial People’s Hospital, University of Electronic Science and Technology of China, Chengdu, China; 2 The MOE Key Laboratory for Neuroinformation, School of Life Science and Technology, University of Electronic Science and Technology of China, Chengdu, China; 3 Brain Cognition and Brain Disease Institute (BCBDI), Shenzhen Institutes of Advanced Technology, Chinese Academy of Sciences, Shenzhen, China; 4 Department of Psychology, Sichuan Normal University, Chengdu, Sichuan China; 5 State Key Laboratory of Brain and Cognitive Sciences, The University of Hong Kong, Hong Kong, China; 6 Department of Psychology, The University of Hong Kong, Hong Kong, China; 7 Department of Psychiatry, University of Cambridge, Hills Rd., Cambridge, CB2 0QQ UK; 8 Department of Psychology, University of Cambridge, Downing St., Cambridge, CB2 3EB UK

**Keywords:** Psychiatric disorders, Psychology

## Abstract

Prosocial and moral behaviors have overlapping neural systems and can both be affected in a number of psychiatric disorders, although whether they involve similar neurochemical systems is unclear. In the current registered randomized placebo-controlled trial on 180 adult male and female subjects, we investigated the effects of intranasal administration of oxytocin and vasopressin, which play key roles in influencing social behavior, on moral emotion ratings for situations involving harming others and on judgments of moral dilemmas where others are harmed for a greater good. Oxytocin, but not vasopressin, enhanced feelings of guilt and shame for intentional but not accidental harm and reduced endorsement of intentionally harming others to achieve a greater good. Neither peptide influenced arousal ratings for the scenarios. Effects of oxytocin on guilt and shame were strongest in individuals scoring lower on the personal distress subscale of trait empathy. Overall, findings demonstrate for the first time that oxytocin, but not vasopressin, promotes enhanced feelings of guilt and shame and unwillingness to harm others irrespective of the consequences. This may reflect associations between oxytocin and empathy and vasopressin with aggression and suggests that oxytocin may have greater therapeutic potential for disorders with atypical social and moral behavior.

## Introduction

Prosocial behavior and aspects of moral behavior (primarily moral emotions, reasoning, and judgment) are closely associated across cultures [1–4], and there is considerable overlap between the brain regions involved [5–7]. Disorders with social behavior problems, such as autism [8], borderline personality disorder [9], psychopathy [10], and obsessive-compulsive disorder (OCD) [11] also tend to show differences in moral behavior compared to neurotypical individuals. However, it is unclear to what extent prosocial and moral behaviors are dissociable and whether different neurochemical systems may be involved.

Serotonin signaling can influence moral judgments [12–15] but also impacts social cognition and motivation and many other behaviors [16]. Similarly, dopamine may play a role in moral decision-making [14, 17] as well as in social motivation and reward [18]. However, greater insight into shared and distinct aspects of mechanisms contributing to prosocial and moral behavior might be achieved by considering neuropeptide systems with more selective effects on social behavior.

The hypothalamic neuropeptides oxytocin (OXT) and vasopressin (AVP) play key roles in social cognition and motivation in both animal models and humans [19, 20] and several clinical trials have reported improved social symptoms in autistic children after their chronic intranasal administration [21–24]. On the other hand, animal models demonstrate that the two peptides can produce opposite effects on stress and anxiety [25–27] and aggression [28, 29], with oxytocin tending to reduce stress, anxiety, and aggression whereas vasopressin can increase them.

Both OXT and AVP have extensive functional interactions with serotonin and dopamine [30–32], but their influence on moral behavior is unclear. Psychiatric disorders with differences in both social and moral behaviors relative to neurotypical individuals tend to have altered blood or cerebrospinal fluid concentrations of OXT [33–36]. Intranasal OXT facilitates emotion processing [37, 38], parochial and/or universal altruism [39, 40], emotional [41–43] and pain [44] empathy, paternal responses to children [45], co-operative behavior [46–49] and generosity [50, 51] as well as punishment for betrayal of trust [52]. Intranasal OXT also influences attraction to individuals with a history of infidelity [53] and reduces jealousy in response partner infidelity [54]. Furthermore, OXT enhances the impact of negative social feedback on learning [55, 56], suggesting that it may increase tolerance to moral transgressions and fear of social punishment. An initial study intranasal OXT only increased endorsements of moral dilemmas involving self-benefit in men but decreased them in women [57]. Other studies found OXT rendered individuals more forgiving of moral transgressions by others [58] and facilitated the speed of acceptance of all types of moral dilemmas and decreased orbitofrontal cortex responses to moral relative to non-moral dilemmas [59]. Moral judgments may also be influenced by OXT receptor genotype [60–62].

Relatively few studies have been conducted on the AVP system and the relationship with altered basal blood or cerebrospinal fluid concentrations in psychiatric disorders with differences in social and moral behavior is less clear [63–65] and in personality disorder, they are associated more with aggression [64]. While, like OXT, AVP facilitates face emotion processing [66–69], attention to social cues [70], and the impact of negative social feedback [55], it does not influence altruism [40], although it may have positive effects on co-operative behavior [49, 71]. While one study reported that AVP, in contrast to OXT, did not influence frontal and basal ganglia regions involved in empathy and reward processing when fathers looked at their children [45], another found it evoked a small increase in empathic concern, but only in individuals who had received higher amounts of paternal warmth [72]. However, animal models have reported that while AVP facilitates male aggression [29], OXT can reduce it [28], and in humans, intranasal AVP may promote aggressive reactions in males [73, 74] or pre-emptive defensive aggression in both sexes [75]. To date, no studies have specifically investigated the effects of AVP on moral behavior.

The current study therefore investigated the effects of intranasal administration of both peptides on two different moral behavior tasks in a cohort of adult male and female subjects (*n* = 180). In line with many studies on moral behavior [76, 77] both tasks focused on responses to scenarios involving deliberate harm to others, with avoidance of harm to others being universal across cultures [78, 79]. The first task uses scenarios with accidental or deliberate harm to others and the subject is required to rate their feelings of self-conscious moral emotions (guilt and shame) to each scenario imagining themselves to be either the agent or a victim (from the EMOTICOM battery) [80]. The second task involves making decisions on whether to endorse actions in scenarios depicting personal or impersonal moral or non-moral dilemmas (from Greene) [81]. Here we specifically focused on responses to personal moral dilemmas involving willingness to directly physically harm one individual in order to save others. Given previous studies showing that OXT influences behaviors related to moral judgment, most notably empathy, we therefore, hypothesized that it should produce stronger effects than AVP on increasing feelings of shame and guilt for causing harm to others and on decreasing willingness to endorse actions inflicting harm on them. Furthermore, given previous evidence for a close relationship between empathy and moral behavior [82] we additionally hypothesized any observed effects on moral emotions would be influenced by trait empathy.

## Materials and methods

### Participants

180 healthy adults (90 males, 18–26 years, mean ± SD age = 20.53 ± 1.88 years) were recruited (For interaction effects in mixed ANOVAs G*Power showed this achieved 84% power in the moral emotions task and 79% in the moral decision-making task – see supplementary for full details). All participants self-reported being free from medical or psychiatric disorders and were within normal range scores on questionnaires for anxiety and depression (see Supplementary Table S1). They were instructed to abstain from alcohol, caffeine, nicotine, or medications for 24 h before the experiment. Female participants were not pregnant or in their menstrual period or taking oral contraceptives. The protocol was approved by the Ethics Committee of UESTC (number 101420210426008) and adhered to the latest revision of the Declaration of Helsinki. Participants provided written informed consent before the start of the experiment and were financially compensated after participation (110 RMB). The study was pre-registered as a clinical trial (https://clinicaltrials.gov/show/NCT04890470).

### Experimental procedure

To control for between-group differences in verbal IQ, mood, clinical symptoms, personality traits, ethical attitudes, validated Chinese questionnaires were completed pre-treatment (see supplementary and Table S1). The Positive and Negative Affective Scale – PANAS [83] and State and Trait Anxiety Inventory – STAI [84] were administered before treatment and after completion of the tasks to measure effects on mood and state anxiety. One-way ANOVAs confirmed the absence of group differences at baseline (see Supplementary Table S1). The Interpersonal Reactivity Index – IRI [85] was administered prior to treatment to permit associations between task performance and trait empathy to be made. Participants were randomized to receive either 24 international units (IU) OXT, 20IU AVP or PLC 45 min prior to the start of two experimental tasks. During this 45-min period participants could read technical magazines or play solitaire card games on a computer to avoid evoking any form of competitive aggression stimulation.

### Treatment administration

All participants were randomly (using a computer-based procedure) allocated into OXT (*n* = 60, 31 males, mean ± SD age = 20.55 ± 1.85 years), AVP (*n* = 60, 30 males, mean ± SD age = 20.13 ± 1.80 years) or PLC groups (*n* = 60, 29 males, mean ± SD age = 20.9 ± 1.95 years). Sterile 24IU OXT (OXT acetate spray supplied by Sichuan Defeng Pharmaceutical Co., Ltd, China) and 20IU AVP (AVP acetate supplied by Bio-Techne China Co., Ltd) doses were administered dissolved in sodium chloride and glycerol. Both were administered as six 0.1 ml puffs, 3 to each nostril interspaced by 30 s. The 24IU OXT dose was chosen since it has been used routinely in many other studies [19] and the 20IU AVP dose was chosen since it is equivalent (i.e. equimolar) to 24IU OXT similar to previous studies [49, 70, 71]. The sterile PLC spray was identical in composition other than the neuropeptide (supplied by Sichuan Defeng Pharmaceutical Co., Ltd, China). Experiments were performed double-blind with spray bottles for the three treatments identical in appearance and coded. The coding was carried out by an individual not involved in the experiment and not revealed until the experiment was completed.

### Moral Emotions task

The Moral Emotions task was from the neuropsychological test battery EMOTICOM [80] but re-programmed via PsychoPy2 [86] with text in Chinese. Experimental stimuli were 14 different cartoon figures depicting moral scenarios with half involving deliberate harm and half accidental harm to others. Cartoon figures were edited to appear more Asian by giving them all black hair and dark eyes. Each cartoon figure was presented twice and participants asked to imagine how they would feel in the situation as either the agent or the victim and give corresponding ratings. Thus, 28 scenarios were displayed randomly to each participant, and feelings of being ashamed, guilty, and feeling “bad” rated when they imagined themselves as the agent, and of annoyance and feeling “bad” when imagining themselves as a victim. A 7-point Linkert scale was used (ashamed, guilty, and annoyed: 1 = not at all and 7 = extremely; feeling “bad”: 1 = feeling bad, and 7 = feeling good). There was no time limitation for watching the cartoons or giving ratings during the task (Fig. S1).

### Moral Judgment task

Twenty-four hypothetical descriptions in Chinese depicting non-moral, moral indirect impersonal harm, and moral direct personal harm scenarios (8 scenarios per condition) were used following a pilot study involving 40 (*n* = 20 female) independent subjects (see Supplementary Methods) from the original 64 scenarios by Greene [71]. In the moral direct personal dilemma scenarios the participant as an agent is asked if they endorsed actions involving causing direct harm to individuals in order to save more whereas for the moral indirect impersonal dilemmas, they were asked if they endorsed indirect actions resulting in saving more individuals from harm. A set of non-moral scenarios were selected as control stimuli. The selected 24 scenarios were correctly classified as representing the specific categories (mean accuracy = 76.15%) (see Supplementary Methods and Table S2 for scenario examples).

The Moral Judgment task was programmed via E-prime 2.0 (Psychology Software Tools, USA). All 24 verbal descriptions were randomly displayed for passive viewing and subsequently paired with a posed question relevant to each scenario (“Is it appropriate to…?”). Participants made forced-choice decisions of either “yes” or “no” to indicate whether they endorsed the choice posed or not and with no time limits (see Fig. S2). At the end of the experiment, participants were presented with all the moral dilemmas again and asked to rate how aroused they felt by them using a Likert scale (from 1 not at all to 7 extremely aroused).

### Statistical analysis

18 participants were excluded (8 in the OXT group, 3 in the AVP group and 7 in the PLC group), due to either technical problems with collecting rating data (*n* = 11), failing to understand instructions properly (*n* = 3), answering insufficient questions (*n* = 2), having a cold (*n* = 1), or taking a medication in the last 24 h (*n* = 1). This resulted in 162 participants being included in the final analysis (OXT = 52 (26 males); AVP = 57 (29 males); PLC = 53 (26 males)). One additional subject was excluded from the Moral Judgement task, due to a data collection technical failure (see Consolidated Standards for Reporting of Trials (CONSORT) flow diagram in Supplementary Fig. S3). The 162 included participants could not identify which treatment they received better than chance (33.3%) after the experiment (48 guessed correctly, χ^2^ = 1.000, *p* = 0.317), confirming successful double-blinding.

All statistical analyses were performed using SPSS 26.0 software (SPSS Inc., Chicago, Illinois, USA). Gender effects were firstly explored using repeated-measures ANOVAs only in the PLC group (see Supplementary and Table S2). Treatment effects were analyzed primarily using repeated-measures ANOVAs and significant interactions explored using Bonferroni-corrected post-hoc multiple comparison analyses within SPSS.

For behavioral indices that showed significant treatment effects, we further explored if they were moderated by trait empathy. Four IRI dimensions of empathy were assessed including perspective taking (PT), fantasy (FS), empathic concern (EC), and personal distress (PD) but only three exhibited acceptable internal consistency and served as moderators (Cronbach’s *α* scores: 0.78 for perspective taking, 0.69 for fantasy, and 0.778 for personal distress). The empathic concern *α* was only 0.58. Separate models were tested for each moderator and behavioral outcome. We estimated the moderation effects using the PROCESS macro for SPSS (Model 1) [87]. Bonferroni corrections were applied for multiple comparisons.

## Results

### Oxytocin, but not vasopressin, increases feelings of shame and guilt

**Fig. 1:**
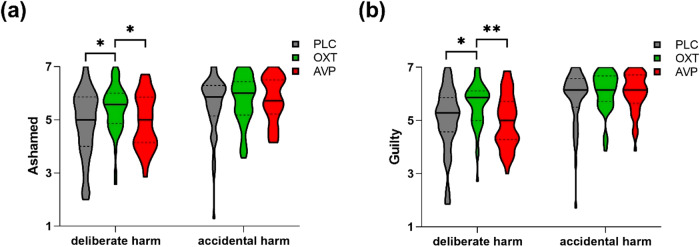
Intention condition x drug effect on moral emotions. Violin plots showing that OXT increased the ratings of feeling **a** ashamed and **b** guilty when deliberately harming others as an agent, relative to both PLC and AVP. * and ** denote significant Bonferroni-corrected post-hoc difference at *p* < 0.05 and *p* < 0.01 res*p*ectively.

For the moral emotions task two-way repeated-measures ANOVAs were used with intention condition (deliberate harm/ accidental harm) as a within-subject factor and treatment (OXT/ AVP/ PLC) as a between-subject factor and ratings (agent - ashamed, guilty and feeling “bad”; victim - annoyed, feeling “bad”) served as dependent variables respectively. For ashamed ratings there was a significant intention condition x treatment interaction (F_(2,159)_ = 5.762, *p* = 0.004, ${\eta }_{p}^{2}$ = 0.068). Post-hoc Bonferroni-corrected tests showed that, in the context of causing deliberate harm to others, OXT significantly increased ashamed ratings relative to both PLC and AVP (OXT vs PLC - *p* = 0.022, Cohen’s d = 0.517; OXT vs AVP - *p* = 0.049, Cohen’s d = 0.519); AVP and PLC did not differ from each other (*p* = 1.000) (Fig. 1a). There were no significant differences between treatments for the accidental harm condition (all *p*s > 0.992).

Similarly, a significant intention condition x treatment interaction was observed for individual ratings of guilt (F_(2,159)_ = 6.860, *p* = 0.001, ${\eta }_{p}^{2}$ = 0.079), due to the intention-specific effect of OXT on deliberate (OXT vs PLC - *p* = 0.012, Cohen’s d = 0.562; OXT vs AVP - *p* = 0.007, Cohen’s d = 0.666; AVP vs PLC - *p* = 1.000) rather than accidental harm (all *p*s > 0.545) (Fig. 1b). Thus overall, OXT but not AVP, increased guilt and shame when participants imagined themselves as agents causing deliberate, but not accidental, harm to others.

For ratings of feeling “bad”, there was no significant intention condition x treatment interaction (F_(2,159)_ = 1.455, *p* = 0.236) or main effect of treatment (F_(2,159)_ = 0.274, *p* = 0.761). However, a main effect of intention condition (F_(1,159)_ = 206.196, *p* < 0.001, ${\eta }_{p}^{2}$ = 0.565) indicated that causing accidental harm as an agent was rated as feeling worse in comparison with causing deliberate harm (*p* < 0.001, Cohen’s d = 1.093) regardless of treatment.

For ratings by subjects as victims of harm there were no treatment-related effects for annoyance (intention condition x treatment interaction: F_(2,159)_ = 0.407, *p* = 0.666; main effect of treatment: F_(2,159)_ = 1.093, *p* = 0.338) or feeling “bad” (intention condition x treatment interaction: F_(2,159)_ = 0.705, *p* = 0.495; main effect of treatment: F_(2,159)_ = 0.084, *p* = 0.919). However, there were significant main effects of intention condition for both annoyance (F_(1,159)_ = 209.62, *p* < 0.001, ${\eta }_{p}^{2}$ = 0.569) and feeling “bad” (F_(1,159)_ = 215.40, *p* < 0.001, ${\eta }_{p}^{2}$ = 0.575) demonstrating that exposure to deliberate harm as a victim was rated more annoying (*p* < 0.001, Cohen’s d = 1.08) and made subjects feel worse (*p* < 0.001, Cohen’s d = 1.207) in comparison to accidental harm, irrespective of treatment.

**Fig. 2:**
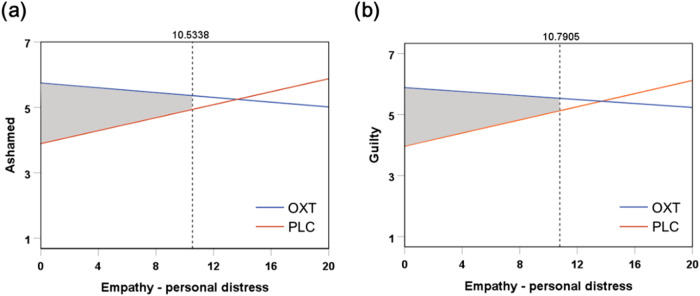
Individual-specific empathy dimension - personal distress level modulated the OXT effect on ashamed and guilty feelings. OXT’s increased effect on ratings of **a** shame and **b** guilt in the context of harming others deliberately was evident when the individual personal distress was below 10.534 (60%; OXT > PLC) and 10.791 (60%; OXT > PLC) respectively. Shaded area identifies regions of significance in which ratings of feeling differ significantly between the OXT and PLC groups at *p* < 0.05. The outer border represents the lowest and highest score of empathy-personal distress subscale in the sample.

A moderation analysis was performed for three dimensions of empathy (perspective taking, fantasy, and personal distress). Treatment (OXT/ PLC) was used as the independent variable and ratings of guilt and ashamed feelings as dependent variables. Separate models were tested for each moderator and Bonferroni corrections for multiple comparisons (i.e. for 3 × 2 = 6 comparisons) were applied. For personal distress, a significant moderation effect was found on both ashamed (*R*^2^ = 0.145, F_(3,101)_ = 5.711, *p* = 0.001, *p*_corrected_ = 0.007) and guilt (*R*^2^ = 0.172, F_(3,101)_ = 7.007, *p* < 0.001, *p*_corrected_ = 0.001) ratings, suggesting that scores on the personal distress dimension moderated the effects of OXT on increasing ashamed (*B* = −0.136, S.E. = 0.046, *T*_101_ = −2.956, *p* = 0.004, *p*_corrected_ = 0.023) and guilt ratings (*B* = −0.140, S.E. = 0.044, *T*_101_ = −3.222, *p* = 0.002, *p*_corrected_ = 0.010; see Table S3) in the context of deliberately harming others. Further disentangling the effect using the Johnson-Neyman method [88] revealed that only when the individual personal distress score was below 60% of the whole sample distribution were treatment group differences (OXT > PLC) for ashamed (Fig. 2a) and guilt ratings (Fig. 2b) considered significant. No significant moderating effects were observed for the other subscales of IRI.

### Oxytocin, but not vasopressin, reduces willingness to endorse dilemmas involving deliberate harm

**Fig. 3:**
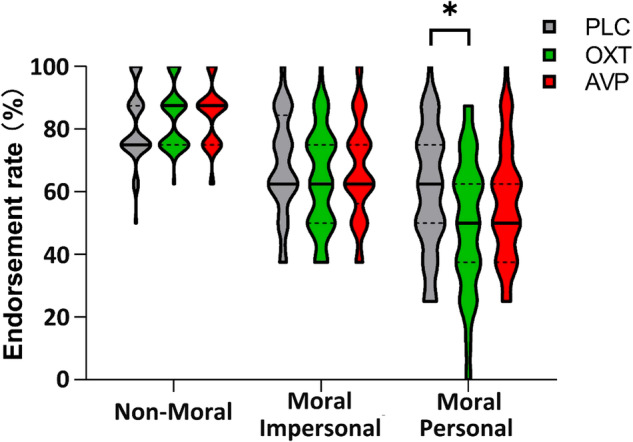
Greene’s taxonomy x treatment effect on moral judgment. Violin plots showing that OXT selectively reduced the endorsement rate towards moral personal scenarios in comparison to PLC. * denotes significant Bonferroni-corrected post-hoc difference at *p* < 0.05.

A 2-way repeated-measures ANOVA with endorsement rate towards scenarios as the dependent variable, Greene’s taxonomy (non-moral/ moral impersonal/ moral personal) as a within-subject factor, and treatment (OXT/ AVP/ PLC) as a between-subject factor yielded a significant taxonomy x treatment interaction (F_(4,316)_ = 2.536, *p* = 0.040, ${\eta }_{p}^{2}$ = 0.031). Post-hoc Bonferroni-corrected tests revealed that OXT selectively reduced endorsements compared with PLC towards moral personal scenarios (OXT vs. PLC - *p* = 0.023, Cohen’s d = 0.510; OXT vs. AVP - *p* = 0.174; AVP vs. PLC - *p* = 1.000) (Fig. 3), while no effect of OXT or AVP was found for moral impersonal (all *ps* > 0.449) or non-moral (all *ps* > 0.412) scenarios, again implying an effect of OXT only in situations involving deliberate harm to others.

For post-test arousal ratings for the different moral dilemmas an ANOVA revealed no significant treatment x taxonomy interaction (F_(4,316)_ = 1.303, *p* = 0.269) or main effect of treatment (F_(2,158)_ = 1.222, *p* = 0.297). However, there was a significant main effect of taxonomy (F_(2,316)_ = 99.865, *p* < 0.001, *η*_p_^2^ = 0.387) indicating that general arousal responses towards the three scenario categories were different from one another, with arousal ratings for moral personal scenarios being the highest and those towards the non-moral scenarios being the lowest (all *p*s < 0.001). Thus, OXT specifically influenced endorsement of personal moral dilemmas involving directly inflicting personal harm but without altering general arousal associated with making decisions.

### Gender and age do not influence treatment-related effects

When both gender and age were included as covariates the results from remained robust (see supplementary Tables S4, S5). A separate ANOVA analysis for the PLC group also revealed no significant gender differences in ratings and choices in the two tasks (see Table S6).

### Oxytocin and vasopressin both reduce post-task negative mood

To examine the potential effects of treatment and the task on mood (both positive and negative PANAS subscales) and state anxiety (SAI), 2-way ANOVAs with time point (pre-test/ post-test) as within-subject factor and drug (OXT/ AVP/ PLC) as between-subject factor were conducted. There was a time point x treatment interaction (F_(2,158)_ = 3.410, *p* = 0.035, ${\eta }_{p}^{2}$ = 0.041) for negative mood, due to reduced negative feelings after both OXT (*p* = 0.002, Cohen’s d = 0.306) and AVP (*p* = 0.006, Cohen’s d = 0.260) treatment but PLC (*p* = 0.772). No significant time point x treatment interactions were found for positive mood (F_(2,158)_ = 2.510, *p* = 0.085) or anxiety scores (F_(2,158)_ = 0.049, *p* = 0.952) (see Table S1). One female subject in the PLC group was excluded since post-test responses were not collected.

## Discussion

In support of our hypothesis, based on previous findings, that social and moral behavior may be dissociable in terms of their neurochemical control we have demonstrated for the first time that OXT but not AVP administration significantly influences moral emotions. Oxytocin specifically increased feelings of shame and guilt when causing deliberate but not accidental harm to others and its effects were greatest in individuals with low trait empathy. Shame and guilt ratings given by our Chinese participants in the EMOTICOM task were similar to those reported in western subjects [80, 89]. We have further demonstrated that OXT specifically reduced endorsements of moral personal choices involving deliberate harm to anyone, no matter what the justification, but without influencing individuals’ general arousal levels. As such, OXT may be acting to strengthen deontological moral decision-making where it is the morality of the action performed rather than its consequence which is important.

This facilitation of deliberate harm aversion following OXT treatment is very much in line with its well-established prosocial and empathic effects [18, 19] and also the strong association between moral and social behaviors [1–4]. Our findings are also consistent with observed decreases and increases in basal cerebrospinal or blood concentrations in psychiatric disorders where moral behavior is atypical [33–36]. While the personal moral dilemmas are more arousing than other types of dilemmas there was no evidence for treatment effects on arousal ratings. A previous study found opposite effects of OXT on the endorsement of self-benefit moral decisions in males and females [57], although in a subsequent study, no gender differences were found and OXT increased the speed with which moral dilemmas were endorsed [59]. Thus, it is possible that there may be some sex differences on the effects of OXT on certain kinds of moral dilemmas.

The effects of OXT on moral emotion ratings revealed that they were moderated by scores on the personal distress subscale of the IRI. Thus, individuals scoring low on the personal distress scale were more likely to exhibit OXT-mediated increases in ratings of shame and guilt. Personal distress is a measure of the affective response to the experiences of others and so OXT appears to mainly facilitate increased harm avoidance in individuals with lower emotional responses to the suffering of others, although only when such suffering is caused by them. This is in line with previous findings that intranasal OXT facilitates emotional empathy in both men and women [41–43, 90] and can increase empathy towards members of an out-group experiencing pain [44].

How might OXT be promoting increased aversion to deliberately harming others? Findings from neuroimaging [7, 91, 92], brain stimulation [93], and brain lesion [94] studies have consistently implicated the medial frontal cortex and its limbic connections with moral emotions and decision-making. Intranasal OXT strengthens functional connectivity between the medial prefrontal cortex and limbic regions [95] and OXT receptor mRNA is decreased in the medial prefrontal cortex in a number of psychiatric disorders where moral behavior can be affected [96]. Oxytocin has extensive functional interactions with diffuse transmitter systems [30–32], notably serotonin, which influence moral decision-making. Increasing serotonin concentrations using serotonin selective reuptake inhibitor (SSRI) drugs such as citalopram increases OXT release [97] and harm aversion in several experimental contexts, including moral decision-making and unfair behavior in economic games [12–15]. Intranasal OXT also alters serotonin receptor activity (5HT1-A) in a number of frontal cortex and limbic brain regions associated with moral decision-making [98]. Furthermore, OXT modulation of the basal ganglia and social reward behavior in animals [99] and its amygdala effects during threat processing in humans [100] are dependent on serotonin signaling. While established OXT interactions with dopamine signaling [101] might also play a role, modulation of dopamine signaling has more of an effect on altruism than harm aversion [14].

The absence AVP effects on either moral emotions or decision-making may reflect it having less of an influence on altruism and empathy and more on risk-taking and aggression [29, 73–75]. Indeed, decreased or increased basal cerebrospinal or blood concentrations of AVP in psychiatric disorders are not consistent with altered moral behavior but may be more associated with levels of aggression [64]. Furthermore, although AVP also interacts with serotonin systems, animal studies suggest this may be more antagonistic, with serotonin-blocking effects of AVP on offensive aggression [102] and SSRIs reducing rather than increasing brain AVP [103]. Thus, while AVP, like OXT, may act to promote a number of prosocial behaviors, only OXT appears to facilitate moral behaviors in terms of harm aversion, possibly reflecting AVP’s more prominent role in offensive aggression.

Several limitations should be acknowledged. Firstly, only single doses of AVP and OXT were used although they were equimolar and the 20IU AVP dose used has both behavioral and neural effects [49, 70, 72–74]. Furthermore, both OXT and AVP reduced post-task negative mood scores in the current study. Secondly, moral emotions and decisions were only made in imagined rather than real-life contexts. Thirdly, participants in the study were all Chinese and there may be some cultural differences. However, moral emotion ratings in the EMOTICOM task are similar in Western subjects [80, 89] and aversion to harming others is similar across cultures [78, 79]. Additionally, OXT has been found to increase empathy across cultures [41–43].

Overall, our findings demonstrate for the first time dissociable effects of two prosocial peptides on moral emotions and judgments, with OXT, but not AVP, specifically enhancing feelings of shame and guilt when imagining causing deliberate harming to others and correspondingly reducing the likelihood that individuals will endorse choosing to do so. This may reflect a greater role for OXT in promoting altruism and empathy and for AVP in promoting aggression and suggests that only OXT may have therapeutic potential for disorders where moral behavior is influenced.

## Supplementary information

Supplementary

## Data Availability

The data that support the findings of this study are available from the corresponding author on reasonable request and with permission of the university administration.
